# The Isolation and Genome Sequencing of Five Novel Bacteriophages From the Rumen Active Against *Butyrivibrio fibrisolvens*

**DOI:** 10.3389/fmicb.2020.01588

**Published:** 2020-07-14

**Authors:** Jessica C. A. Friedersdorff, Alison H. Kingston-Smith, Justin A. Pachebat, Alan R. Cookson, David Rooke, Christopher J. Creevey

**Affiliations:** ^1^Institute of Biological, Environmental and Rural Sciences (IBERS), Aberystwyth University, Aberystwyth, United Kingdom; ^2^Institute for Global Food Security (IGFS), Queen’s University, Belfast, United Kingdom; ^3^Dynamic Extractions Ltd., Tredegar, United Kingdom

**Keywords:** bacteriophage, rumen, lytic phages, phage genome, Butyrivibrio phage, *Butyrivibrio fibrisolvens*

## Abstract

Although the prokaryotic communities of the rumen microbiome are being uncovered through genome sequencing, little is known about the resident viral populations. Whilst temperate phages can be predicted as integrated prophages when analyzing bacterial and archaeal genomes, the genetics underpinning lytic phages remain poorly characterized. To the five genomes of bacteriophages isolated from rumen-associated samples sequenced and analyzed previously, this study adds a further five novel genomes and predictions gleaned from them to further the understanding of the rumen phage population. Lytic bacteriophages isolated from fresh ovine and bovine fecal and rumen fluid samples were active against the predominant fibrolytic ruminal bacterium *Butyrivibrio fibrisolvens*. The double stranded DNA genomes were sequenced and reconstructed into single circular complete contigs. Based on sequence similarity and genome distances, the five phages represent four species from three separate genera, consisting of: (1) Butyrivibrio phages Arian and Bo-Finn; (2) Butyrivibrio phages Idris and Arawn; and (3) Butyrivibrio phage Ceridwen. They were predicted to all belong to the *Siphoviridae* family, based on evidence in the genomes such as size, the presence of the tail morphogenesis module, genes that share similarity to those in other siphovirus isolates and phylogenetic analysis using phage proteomes. Yet, phylogenomic analysis and sequence similarity of the entire phage genomes revealed that these five phages are unique and novel. These phages have only been observed undergoing the lytic lifecycle, but there is evidence in the genomes of phages Arawn and Idris for the potential to be temperate. However, there is no evidence in the genome of the bacterial host *Butyrivibrio fibrisolvens* of prophage genes or genes that share similarity with the phage genomes.

## Introduction

Despite the abundance of viruses in the rumen, little is known about the bacteriophage (phage) population in the rumen microbiome compared to other predominant microbes in this environment (Gilbert et al., 2020). The influences that phages have on ruminal function and digestion are yet to be understood (Anderson et al., 2017). Early studies based on electron microscopy observations of the viral fractions of rumen fluid (Hoogenraad et al., 1967; Paynter et al., 1969; Klieve and Bauchop, 1988) established that a highly diverse phage population exists in high abundance in ruminal fluid and could influence bacterial populations (Orpin and Munn, 1973). It is only now with the application of metagenome sequencing to rumen associated viruses that the full extent of the phage community in this environment is beginning to be uncovered and better understood (Berg Miller et al., 2012; Gilbert et al., 2017).

Rumen phages are those that either target rumen bacterial species, or those that are believed to reside in the rumen, evidenced either by direct sampling of rumen fluid (Klieve and Bauchop, 1988), or though isolation from ruminant associated samples, such as from compacted manure from transport trucks, and abattoir kill-floor run-off (Klieve et al., 2004). Indeed, samples from these two rumen-associated environments (as well as from sewage samples) yielded five phages that are, to date, the only genomes of lytic rumen phages to be sequenced and published in the context of the rumen virome. Of these five, three belonging to the *Siphoviridae* family; two that target the bacterial host *Prevotella ruminicola* and one which targets *Streptococcus bovis*, and another two belonging to the *Podoviridae* family that target *Ruminococcus albus* (Gilbert et al., 2017). These seminal efforts applied and adapted methods for isolating and characterizing lytic phages that infect anaerobic bacteria known to reside in the rumen. However, there is still much to be learned about phages in this environment and the opportunity exists to further investigate their presence in samples taken directly from rumen fluid and fresh feces, as well as exploring phages that target other rumen bacterial hosts.

Not only are individual rumen phages of interest, but the application of metagenomic sequencing on the viral fraction of rumen fluid offers a more holistic approach to observing rumen phage populations. For example, metagenomic analysis of the rumen viromes of three cattle indicated that the phage community is formed of twice as many temperate phages as lytic phages, with the bacterial hosts mostly belonging to the *Firmicutes* and *Proteobacteria* phyla (Berg Miller et al., 2012). Additionally, it was concluded that because the abundance of phage populations tended to correlate with the abundance of the phyla of their prospective bacterial hosts, phages play an important role in the rumen microbiome, manipulating the gene pool through the genetic transfer activity of temperate phages, and bacterial population control through cell lysis activity of the lytic phages (Berg Miller et al., 2012). Another metagenomic study of the viral populations in the rumen of steers fed different diets revealed that although there are changes in viral communities with a change in diet, there were abundant populations of phages found across all samples, indicating a core virome (Anderson et al., 2017). An analysis of DNA viruses in buffalo revealed the order *Caudovirales*, the tailed bacteriophages, formed the largest community, and within this the *Siphoviridae* and *Myoviridae* families were predominant (Parmar et al., 2016). A similar study of rumen fluid from sheep and goats also revealed that of the reads that could be identified, the highest proportion shared similarity to the *Siphoviridae* family, yet the majority of the reads were unidentified (Namonyo et al., 2018). Without representative genomes of the rumen phage population, the ability to make statements about anything more detailed than the viral family level is limited. Therefore, the novel isolation and sequencing of cultured phages from rumen-associated samples, in particular rumen fluid, would be of huge benefit to the research community.

This study presents five novel bacteriophages directly isolated from ovine and bovine rumen fluid and fresh fecal samples, along with their sequenced and annotated genomes. To date, this is the second report of phage genomes to be sequenced from rumen associated samples, and the first to report sequence data of isolated and cultured phages against the bacterial host *Butyrivibrio fibrisolvens*. Previously, filamentous phage-like particles were observed in cultures of *B. fibrisolvens* AR14 cultures when induced with mutagen mitomycin C (Klieve et al., 1989), suggesting the induction of a prophage, but these were not cultured or explored further. As a predominant bacterium in the rumen, *B. fibrisolvens* is thought to contribute to a range of functions, as it has shown abilities such as fiber degradation and xylan fermentation (Cotta and Hespell, 1986), proteolysis (Sales et al., 2000) and biohydrogenation (Kepler et al., 1966). *B. fibrisolvens* has also been isolated from human fecal samples (Brown and Moore, 1960; Rumney et al., 1995), which was shown to be related to ruminal strains and is thought to play a role in butyrate production from starch degradation in the human colon (Barcenilla et al., 2000; Ramsay et al., 2006).

## Materials and Methods

### Bacterial Culturing Methods

*Butyrivibrio fibrisolvens* DSM 3071 was cultured and maintained using M2 medium (Hobson, 1969); using 10 ml/L sodium lactate solution at 60% (w/v), tryptone (Melford, Ipswich United Kingdom) as the protein source, and minerals (b) consisting of KH_2_PO_4_, 3.0 g/L; (NH_4_)_2_SO_4_, 6.0 g/L; NaCl, 6.0 g/L; MgSO_4_⋅7H_2_O, 0.6 g/L; CaCl_2_⋅2H_2_O, 0.6 g/L.

### Rumen-Associated Samples

Samples of rumen fluid and feces were obtained from fistulated sheep and cows. Hand grabs of rumen contents were obtained through the cannulae of three 7-year-old Aberdale cross Texel sheep which were grazing *ad libitum* on grass, with ∼300 g sugar beet and grass nuts supplemented each morning. Rumen fluid samples were obtained in the same way from three 10-year-old Holstein-Friesian dry cows which were grazing *ad libitum* on grass, with ∼500 g dairy concentrates in the morning during sampling. Rumen fluid was obtained under the authority of licenses under the United Kingdom Animal Scientific Procedures act 1986. All animal associated research is managed according to the Aberystwyth University Animal Welfare and Ethics Review Board^1^. Each of the sheep rumen fluid samples were squeezed and strained through a sieve and then combined into one air-tight pre-warmed container. The same procedure was carried out for the cow rumen fluid and collected in another air-tight pre-warmed container.

Feces were caught in a gloved hand once excreted naturally from three sheep of the same flock and transferred to sterile plastic bags. Cow feces were obtained soon after excretion; a small amount of fresh fecal matter was taken from the field, collected into pots, and transferred into sterile centrifuge tubes. Three fecal samples each for cows and sheep were collected this way and screened for phage presence. Where necessary, fecal samples were refrigerated for less than 1 week before use to avoid a freeze/thaw cycle if stored in a freezer.

### Screening Phage Filtrates From Rumen Fluid and Feces

For both sheep and cows, phage filtrates were made from both the rumen fluid and fecal samples as per the methods described previously (Klieve, 2005; Klieve and Gilbert, 2005). The rumen fluid was aliquoted into centrifuge tubes, spun at 15,000 × *g* for 15 min at 4°C, and the supernatants stored on ice and filtered through 0.45 μm pore size low-protein binding PES syringe filters and retained on ice. Around 10 g of feces were weighed out into centrifuge tubes and a volume (in ml) equivalent to the mass (in g) of the sample of Phage Storage Buffer [PSB; 20 mM Tris–HCl, 200 mM NaCl, 20 mM MgCl_2_, 0.1% (w/v) gelatin (Klieve, 2005)] was added and incubated at room temperature for 1 h with gentle mixing on a tilting laboratory shaker. After spinning at 15,000 × *g* for 15 min at 4°C, the supernatant was filtered through a 0.45 μm pore size syringe filter. All filtrates were wrapped in foil and stored at 4°C.

Viral particles were concentrated from filtrates (and later lysates) by the addition of polyethylene glycol (PEG) and sodium chloride (NaCl) with modifications to methods published previously (Bourdin et al., 2014; Antibody Design Laboratories, 2015; Gutiérrez et al., 2018; Namonyo et al., 2018). To phage solutions, PEG 8000 and NaCl was added to a final concentration of 10% (w/v) and 0.5 M, respectively, and used to precipitate phages overnight at 4°C. Concentrated phages were collected by spinning at 12000 × *g* for 30 min at 4°C. The supernatant was discarded, and the pellet resuspended in a smaller volume (a tenth of the initial volume) of suitable phage buffer, wrapped in foil and refrigerated until further use. Previous studies have shown phage filtrates to remain viable for months or even years at 4°C (Klieve, 2005).

A lawn of host bacteria was achieved by adding 1 ml of overnight (or actively growing) culture of *Butyrivibrio fibrisolvens* DSM 3071 to 3 ml of warm 0.8% (w/v) M2 agar (soft agar), mixing and pouring over set bottom agar [1.5% (w/v) M2 agar] in the anaerobic cabinet with gas mix of 10:10:80 of CO_2_, H_2_ and N_2_ (Whitley A35 Anaerobic Workstation, Don Whitley Scientific) (Klieve, 2005). Once set, a spot test was carried out by spotting 10 μl of each phage filtrate in triplicate onto the host lawn and incubating for >24 h at 39°C, or until adequate lawn growth [similar method that was used by Nale et al. (2016)]. Any resulting areas of lysis or clearing on the bacterial lawn was indicative of phage activity. Positive spots or plaques were scraped using a 5 μl inoculating loop, extracting top soft agar, and mixed well in a small volume of PSB, vortexed briefly and left to stand at room temperature for 30 min and stored at 4°C until testing again on the same host. Where a sample spot contained what appeared to be a very small area of lysis or that resembled a small plaque, these were picked using a pipette tip and all combined into a microcentrifuge tube containing a small volume of PSB, vortexed briefly and left to stand as above, before undergoing further plaque assays. Combining putative plaques in this way from a variety of samples increases the likelihood of phage isolation and reduces consumable use but removes the traceability of subsequent phage isolation to the original fecal or rumen fluid sample, which for this study was deemed acceptable.

### Isolation and Purification of Bacteriophages

Upon an initial indication of phage activity in a sample, then in later stages to purify and propagate positive phage samples, a plaque assay was carried out. An aliquot of 10 μl of the sample positive for phage activity was combined with 1 ml of host bacterial culture, left to incubate for no more than 15 min, mixed with 3 ml of soft agar [0.8% (w/v)] and poured over bottom agar [1.5% (w/v) M2 agar] (Klieve, 2005). Plaques with clearly defined, non-overlapping zones of clearance were picked using a pipette tip, placed into a small volume of PSB (∼200 μl) vortexed briefly, left standing for half an hour at room temperature, and tested again. Dilutions of the picked plaques were made using PSB to avoid confluent growth in the early stages of purification, so that single isolated plaques with one morphology type could be observed.

Once the plaque morphology was believed to be homogenous, or at least three rounds of purification had taken place, the phage was eluted from a confluent plaque assay plate by adding 5 ml of PSB to the plate, macerating the agar gently using a spreader, and leaving to incubate at room temperature on a rocker, for at least 30 min. The eluate was aspirated using a pipette tip into a microcentrifuge tube, spun at 15,000 × *g* for 2 min to pellet any agar, and the supernatant was filtered through a 0.45 μm pore size PES syringe filter. This resulted in five phage samples, temporarily termed phage C, D, J, M, and P.

### Nucleic Acid Extraction and Sequencing

Phages from 800 μl of eluates were precipitated using PEG/NaCl overnight and resuspended in a volume one tenth of the initial volume, 40 μl of which was then used for nucleic acid extraction. Controls were added to the nucleic acid extraction process to evaluate the extraction process and other downstream applications, such as gel electrophoresis. The controls were comprised of molecular grade water as the negative control, and a “host lysate sample” as a positive control, which was made by taking an aliquot of host bacterial liquid culture, vortexing briefly and boiling at 100°C for 15 min to lyse the cells, before spinning at 15000 × *g* for 10 min, and filtering the supernatant through a 0.45 μm pore size PES syringe filter. Of this, 600 μl was precipitated overnight with PEG/NaCl, as well as the negative control. From each of these controls, 40 μl also underwent DNA extraction alongside the phage samples. The FastDNA^TM^ Spin Kit for Soil (MP Biomedicals, Solon, OH, United States) was used to extract DNA from the lysate samples, using the proprietary FastPrep-24 for 30 s at 6.0 m/s, and spinning at 14,000 × *g* for 5 min at 18°C. DNA was eluted from the column with 30 μl of supplied DES water. The DNA concentration was determined using the Qubit fluorometer (Qubit 3 Fluorometer, Invitrogen by Thermo Fisher Scientific) and the high sensitivity DNA assay. Those samples that had a low concentration were increased using a DNA Speed Vac (DNA 100, Savant), or in the case of phage sample J, was re-extracted using 900 μl of phage eluate and combining this with 300 μl of the supplied sodium phosphate buffer in the first step. DNA was eluted from the column first with 30 μl of DES water, then a second time with 50 μl. The concentration of DNA in the re-extracted and concentrated sample were measured again, and all samples were diluted with nuclease free water to achieve a final concentration of ∼2 ng/μl in 10 μl necessary for sequencing. Quality of the DNA was tested using spectrophotometry and the 260/280 ratio (Epoch, BioTek Instruments) and sequencing was done in-house, where the isolated phage DNA was first diluted to 1 ng/ul, and libraries then prepared using the Illumina NextEra XT protocol as per the manufacturer’s instructions (selecting AMPure bead ratio as suggested for 2 × 300 bp reads). Libraries were quantified via Qubit fluorescence spectrophotometry, pooled at an equimolar ratio, and diluted to 6 pM before loading on an Illumina MiSeq platform using a v3 600-cycle kit in 2 × 300 bp format. The five samples are available on NCBI as BioSamples under the BioProject accession code PRJNA613207, and raw reads for these in the Sequence Read Archive (SRA) under the accession code SRP255162.

### Quality Control and Assembly

Quality control of the raw reads was carried out using FastQC v.0.11.8 (Andrews, 2010), followed by quality trimming using the paired end default settings in Sickle (Joshi and Fass, 2011) and assembly using SPAdes v3.13.0 on paired and single reads after trimming, with just “assembly-only” option applied (Bankevich et al., 2012), as conducted for phage genomes previously (Rihtman et al., 2016; Sazinas et al., 2018). Contigs were visualized using Bandage v0.8.1 (Wick et al., 2015), and those circularized contigs with the highest coverage for assembled reads of a reasonable length (>10 kbp) were extracted as the phage genomes. The phage genome contigs were visualized manually using Geneious Prime 2020.1.2^2^ and repeat regions were identified. A 127 bp length of sequence in each of the genomes was repeated as an overlap of the assembled start and end (as the genomes were circular), which corresponded to the k-mer size used by SPAdes during assembly, and was removed. BWA-MEM v0.7.16a-r11810 (Li, 2013) was then used with default settings to align all reads in a sample to all of the corresponding assembled contigs, and SAMtools v1.5 (Li et al., 2009) was then used to extract the alignments to the phage contig and manage output files. Coverage was assessed using the depth command in SAMtools and calculating the coverage for the entire genome by averaging the coverage for each base in a genome.

The quality of genomes was assessed using Pilon v1.23 (Walker et al., 2014), and then were rearranged using the terminase gene, which was predicted using Prokka v1.12 (Seemann, 2014), supplemented with the Caudovirales database^3^. The genomes were reordered and orientated manually using Geneious Prime, such that the start of the linear sequence for the circularized genome was at the first base of the first gene found downstream of the terminase gene, as recommended previously (Russell, 2018).

### Comparing and Annotating the Phage Genomes

The entire genome sequences were queried against the viruses (taxid:10239) nucleotide collection (nr/nt) using blastn (Zhang et al., 2000) and default settings (carried out on 15/05/2020). For comparative genomics, average nucleotide identity (ANI) was calculated using FastANI v1.3 (Jain et al., 2018) using default settings. Genomic synteny was visualized using ProgressiveMauve within Geneious Prime, using default settings.

Open reading frames (ORFs) were then predicted using Glimmer v3.02 (Delcher et al., 2007) with default settings, GeneMarkS-2 (Lomsadze et al., 2018) with Prokaryotic sequence type, genetic code 11 and GFF3 output using the online tool^4^, Prodigal v2.6.3 with default settings with output as GFF (Hyatt et al., 2010) and PHANOTATE v1.2.2 (McNair et al., 2019) with default settings. All ORFs were manually curated using Geneious to visualize all predicted ORFs, allocating each putative ORF into one of four groups; (A) those ORFs where all gene callers agreed with presence, start and end; (B) those ORFs where all gene callers agreed with gene presence, but with different predicted starts and/or ends; (C) those ORFs where the majority of gene callers agreed on ORF presence, with same start and ends; (D) those ORFs where the majority of gene callers agreed on ORF presence, but with different predicted starts and/or ends.

Nucleotide and protein sequences for ORFs in all four categories were obtained using gff2bed tool [Bedops; v2.4.37; (Neph et al., 2012)], getfasta [Bedtools; v2.27.1; (Quinlan and Hall, 2010)] and transeq [Emboss; v6.6.0.0; (Rice et al., 2000)]. These were then annotated by searching for homologs with BLAST v2.8.1+ (Altschul et al., 1990; Camacho et al., 2009) and all BLAST hits that achieved an e-value greater than 10^–5^ (Aziz et al., 2018) and query coverage >80%, as used by Prokka (Seemann, 2014) were retained. Custom nucleotide and protein databases comprised of a number of NCBI databases were used, including representative viral reference genomes^5^, viral genomes, proteins and non-redundant proteins from the 8th July 2019 RefSeq database^6^, against which the full genomes and nucleotide gene sequences were searched; alongside the Caudovirales database^3^ and the prokaryotic virus orthologous groups (pVOGs) database supplied with multiPhATE v1.0 (Zhou et al., 2019). Protein databases were obtained from Swissprot^7^ and virus specific orthologous groups from EGGNOG^8^, annotating the best hits with UniProt IDs. Each ORF for each genome was manually curated and annotated using the best of the retained hits, where multiple hits with equally good scores from different databases were all recorded. The name of the genome containing the gene with the best hit(s) for each ORF was also noted. Where gene callers did not agree on an ORF, the ORF with the best hit was used, considering coverage of query and subject and percentage identity to determine the best.

Protein motifs were also predicted using hmmscan in HMMER v3.1b2 (Eddy, 2011) against pfam^9^, TIGRfam^10^ (Haft et al., 2003) and HAMAP^11^ (Lima et al., 2009) databases. A consensus gene name was then derived manually for each predicted ORF for each phage genome based on the evidence gathered from homology searches. Numbers of overlapping genes were obtained from the GFF files for each of the genomes, and tRNAs were predicted using tRNAscan-SE v2.0^12^ assuming a bacterial sequence source and otherwise default settings (Chan and Lowe, 2019) and added to the annotated ORFs. All ORFs were also allocated a functional category, for example Structural or Lysis, depending on the process(es) to which the predicted gene contributes. ORFs that that were not assigned an annotation were allocated to the Hypothetical category, whereas those that shared sequence similarity with another hypothetical gene in another phage genome were allocated to Uncharacterized.

TMHMM v2.0 (Krogh et al., 2001) was run to detect transmembrane regions, einverted was used in Emboss to find inverted repeats and PHACTS^13^ (McNair et al., 2012) to determine lifestyle of the phage. Promoters were identified in each genome using PhagePromoter^14^ (Sampaio et al., 2019) setting the lifecycle for each phage with the outcome of PHACTS, assuming all phages were in the *Siphoviridae* family, host bacterial genus as other, both directions and a threshold of 0.5. Terminators were predicted using FindTerm^15^ (Solovyev and Salamov, 2011), showing all putative terminators with energy threshold value -10, as recommended previously (Aziz et al., 2018). Promoters and terminators were then manually checked, removing terminators present within genes whilst retaining ones present in intergenic regions or in the 3′ end of upstream genes (Aziz et al., 2018), and choosing promoters in the correct orientation and with the highest score, where multiple were present.

### Phylogenetics and Phylogenomics

The nucleotide genome sequences of these genomes were uploaded into ViPTree (Nishimura et al., 2017) using default settings, dsDNA nucleic acid type, Prokaryote host categories and a user-defined gene annotation file. From the resulting tree from the ViPTree analysis, the closest related taxa were identified as those in the same clade as the Butyrivibrio phage genomes, of which there were 13, and were downloaded using the NCBI ID supplied by ViPTree. The nucleotide sequences of these 13 genomes, along with the five phage genomes from this study, were then analyzed using the Genome-BLAST Distance Phylogeny (GBDP) method (Meier-Kolthoff et al., 2013) with default settings recommended for prokaryotic viruses in VICTOR^16^ (Meier-Kolthoff and Göker, 2017) using the D0 formula.

### Assessing Host Interactions

The host genome of the bacterial host *Butyrivibrio fibrisolvens* DSM 3071 was downloaded (NCBI Reference Sequence NZ_FQXK01000003.1) and the GC content and codon usage were summarized in Geneious Prime. Additionally, the presence of prophages was predicted using the PHASTER online tool^17^ (Zhou et al., 2011; Arndt et al., 2016). The amino acid sequences of the predicted prophage genes were subjected to a sequence similarity search using BLASTP against the protein sequences for genes predicted in the five phage genomes from this study and filtered using the same thresholds as previous. CRISPR/Cas genes were identified in the reference genome and in 497 genomes of the Hungate1000 collection (Seshadri et al., 2018) using CRISPRCasFinder (v4.2.19) with default settings (Couvin et al., 2018). The resulting CRISPR spacer sequences were queried against the phage genomes from this study using blastn with default settings.

## Results and Discussion

The screening of bacteriophages from a total of eight rumen-associated samples resulted in the isolation of five phage samples active against *Butyrivibrio fibrisolvens* DSM 3071. The implementation of PEG/NaCl to concentrate bacteriophages from fecal and rumen fluid phage filtrates resulted in the observation of plaques which were further purified to create five phage samples that were seemingly homogenous and morphologically distinct in plaque assays. These were informally labeled as samples C, D, J, M, and P, and correspond to the BioSamples deposited in the NCBI database. From these phage samples, DNA was successfully extracted using a bead beating method and sequenced. After quality control, trimming and assembly, each of the five samples yielded one fully reconstructed circularized phage contig, except for phage sample J, which yielded two contigs each with different genome lengths and coverage. The reconstructed contigs surpassed the recommended minimum of 100-fold coverage recommended for phage genomes (Russell, 2018).

Further analysis of these phage genomes revealed that the phage genome reconstructed from sample J (J-1) with the highest coverage was 100% similar at the nucleotide level to the phage genome reconstructed in phage C sample, as determined by ANI. This genome did not undergo further analysis. The whole phage genomes were queried in the NCBI virus nucleotide database and revealed that there was no significant similarity to viral genomes published and deposited in this database previously. Further searches against the viral representative reference sequence database and viral non-redundant RefSeq database from NCBI also did not reveal similarity to any previously deposited viruses. Therefore, as it is likely that these five phage genomes are novel, they have been named; phage sample C yielded Butyrivibrio phage Bo-Finn, phage sample D yielded Butyrivibrio phage Arawn, phage sample J-2 yielded Butyrivibrio phage Idris and phage sample M yielded Butyrivibrio phage Ceridwen. The summary of the samples, the source of the samples, the genome contigs reconstructed and subsequent names can be seen in Table 1.

**TABLE 1:** Summary table of phage sample and its source, the genomes constructed, coverage and allocated phage name.

Phage Sample	Source	Genome Contigs Reconstructed	Average Coverage Over Entire Genome	Butyrivibrio phage
J	Sheep	J-1	1399.46	N/A
	rumen fluid	J-2	242.56	Idris
C	Cow rumen fluid	C	125.832	Bo-Finn
P	Cow feces	P	754.716	Arian
D	Mixture*	D	1390.5	Arawn
M	Mixture*	M	352.867	Ceridwen

*Mixture – plaques from one sample of cow feces, cow rumen fluid and sheep rumen fluid were combined and tested again to produce the plaques from which these phages were isolated.

**FIGURE 1:**
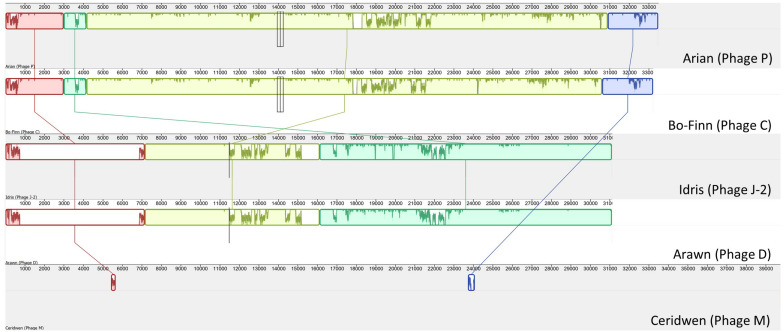
Mauve alignment (Darling et al., 2004) of the five Butyrivibrio phages. Colinear blocks are color-coded, with the similarity graph plotted within the block and lines join similar blocks across genomes.

To determine the similarity between these phage genomes, genome comparisons were first carried out using Mauve (Figure 1), then by calculating the ANI between any two genomes that appeared similar. As is apparent in Figure 1, Butyrivibrio phage Arian and Butyrivibrio phage Bo-Finn share a similar genome length and genome synteny, and 98.6% ANI. Butyrivibrio phage Arawn and Butyrivibrio phage Idris also share a similar genome length and genome synteny to each other, and 98.96% ANI.

The reconstructed phage genomes ranged from 31 (Arawn) to 39.7 Kbp (Ceridwen) in length (Table 2), with numbers of predicted open reading frames (ORFs) ranging from 50 (Arawn) to 73 (Ceridwen). The percentage of ORFs with homology to known sequences ranged from 40% (Ceridwen) to 52% (Arawn) (Table 2). All phage genomes have overlapping genes, ranging from 15% of genes in Idris, which has the smallest genome, and up to 30% of the genes in phage Ceridwen, which has the largest genome. Interestingly, this correlation is opposite to that seen across other viruses, where smaller viral genomes were generally found to have more overlapping genes (Brandes and Linial, 2016). All genomes have small intergenic regions, with few areas of the genome that are not populated by ORFs, and many ORFs overlap, characteristics which are common in phage genomes (McNair et al., 2019).

**TABLE 2:** Genome statistics summary.

Butyrivibrio phage	Genome Length (bp)	GC (%)	ORFs	ORFs with best hit homologous proteins	Most common phage name (number of best hit homologous proteins)*
Arawn	31118	46.9	50	26	Paenibacillus phage PG1 (2)
Arian	33499	39.7	54	25	N/A
Bo-Finn	33227	39.7	51	24	N/A
Ceridwen	39745	40.8	73	29	Clostridium phage phiCP13O (2), Clostridium phage phiCP26F (2)
Idris	31128	46.9	55	25	Paenibacillus phage PG1 (2)

*Using the most common phage name with the number of best hit homologous proteins was a method used previously with rumen phages (Gilbert et al., 2017). N/A indicates that no single phage genome shared more than one best hit homologous protein.

### Phages Bo-Finn and Arian

**FIGURE 2:**
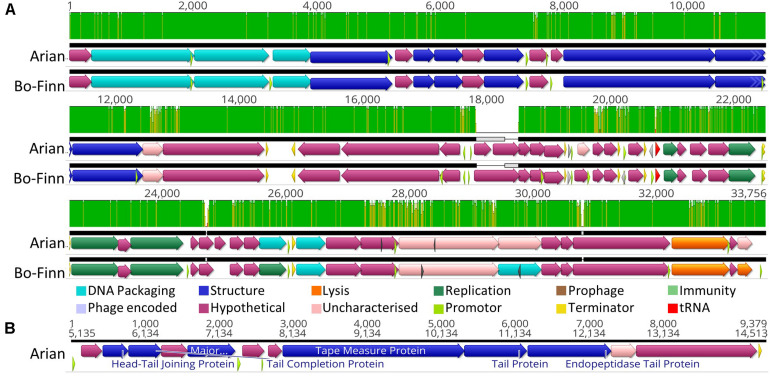
Analysis of the Butyrivibrio phage Arian and Bo-Finn genomes. **(A)** Mauve alignments (Darling et al., 2004) of the two genomes showing annotated ORFs, with directionality and function according to color. Nucleotide similarity is expressed as a graph, with green indicating 100%, yellow > 30%, red < 30%. **(B)** The organization of the tail morphogenesis module in phage Arian. Uncharacterized proteins indicate where the protein was homologous to one reported previously, but had an unknown function, whereas hypothetical proteins did not show similarity above the set thresholds to any known proteins. Phage encoded proteins are those where there was uncertainty about their function.

Butyrivibrio phages Bo-Finn and Arian are different isolates that belong to the same species and genus. A score of 98.6% ANI is greater than the 95% nucleotide sequence identity threshold indicative of the same species, as set by the International Committee on Taxonomy of Viruses (ICTV) (Adriaenssens and Brister, 2017). The two genomes are syntenous and share high sequence similarity across their entire genome (Figure 2A). The most pronounced difference is around 18 Kbp, where a subsequence is present in each genome that is not found in the other genome, as shown by the gray boxes in Figure 2A. Both phages have tail genes that correspond to those found in most members of the *Siphoviridae*; tail terminator, major tail protein, two chaperones, tape measure protein (TMP), distal tail protein, tail-associated lysozyme and baseplate/tip proteins, which together form a functional ‘tail morphogenesis module’ (Veesler and Cambillau, 2011). Figure 2B shows these genes in Butyrivibrio phage Arian, including a promoter and terminator, suggesting that these genes can be regulated together. Not all genes here showed homology to previously identified proteins, but if the tail morphogenesis module is conserved, these hypothetical proteins may be involved in the tail formation. Additionally, of the predicted ORFs that were homologous to genes in other phages, 10 in the Bo-Finn genome and 11 in the Arian genome shared homology to phages of the *Siphoviridae* family, six in each genome to phages of *Myoviridae*, and one each to a phage of *Podoviridae*. Along with the absence of any tail sheath proteins which are common to *Myoviridae* (Adriaenssens and Cowan, 2014), and the majority of genes being similar to other *Siphoviridae* phages, it is likely that these two phages belong to the *Siphoviridae* family.

Butyrivibrio phage Bo-Finn was predicted by PHACTS non-confidently to undergo the temperate lifecycle, whereas Arian was predicted non-confidently to undergo the lytic lifecycle. This suggests that there was some uncertainty with predicting the lifecycle using the proteomes of these two phages and that this outcome was not statistically supported (McNair et al., 2012). The major capsid proteins in these two phage genomes were predicted using protein motifs, resembling those in the HK97 family of major capsid proteins, populated by temperate phages (Haft et al., 2003). This suggests that these phages could undergo the temperate lifecycle, yet there is no further evidence in the genome to suggest this ability; no immunity repressors, partitioning genes or integration and excision enzymes were annotated, which are common functions associated with temperate phages (Mavrich and Hatfull, 2017; Stark, 2017). Additionally, these two phages were observed to undergo the lytic cycle *in vitro* and phage Arian harbors an endolysin (QHJ73651.1), and phage Bo-Finn an endolysin (QHJ73713.1) and putative holin (QHJ73715.1). Moreover, a tRNA gene for the amino acid glutamine was predicted in these genomes, which is used in 3.6% of coding genes in genome of phage Arian and 4.4% in Bo-Finn, compared to 2.7% of coding genes in the bacterial host genome (Supplementary Table S1). These codon usage statistics suggest that this codon is utilized more often in the phage genome than the host genome and therefore encoding this tRNA would increase efficiency and virulence, something that has been noted previously in lytic phages (Bailly-Bechet et al., 2007). Because these genomes are so highly similar, it is likely that they would both undergo the same lifecycle, which is not reflected in the PHACTS results. Evidence so far would therefore indicate that these phages are virulent, however, with some ORFs remaining uncharacterized, there may be novel proteins associated with the temperate lifecycle. The full annotated genomes can be seen for phage Arian and Bo-Finn in Supplementary Figures 1B,C, respectively.

A blastn search for the entire genome sequences of phage Arian and Bo-Finn against the NCBI virus genome database did not reveal any significant alignments covering more than 8% of the query genome.

### Phages Arawn and Idris

**FIGURE 3:**
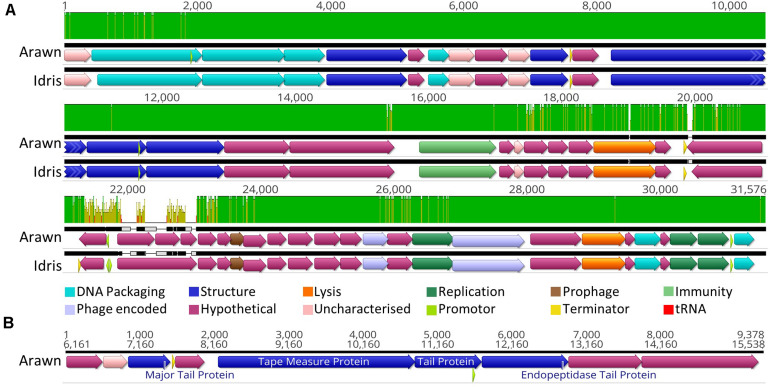
Analysis of the Butyrivibrio phage Arawn and Idris genomes. **(A)** Mauve alignments (Darling et al., 2004) of the two genomes showing annotated ORFs, with directionality and function according to color. Nucleotide similarity is expressed as a graph, with green indicating 100%, yellow > 30%, red < 30%. **(B)** The organization of the tail morphogenesis module in phage Arawn. Uncharacterized proteins indicate where the protein was homologous to one reported previously, but had an unknown function, whereas hypothetical proteins did not show similarity above the set thresholds to any known proteins. Phage encoded proteins are those where there was uncertainty about their function.

Butyrivibrio phages Arawn and Idris also share high sequence similarity (98.96% ANI). The two genomes are syntenous, with highly similar genome sequences at the nucleotide level. The most noticeable difference between the two genomes is at 21.3 Kbp, where the similarity is low, and a different sequence in phage Arawn results in three ORFs predicted in this region (Figure 3A). The series of tail proteins present in Arawn (Figure 3B) also resembles the tail morphogenesis module common to phages in the *Siphoviridae* family (Veesler and Cambillau, 2011). Unlike phage Arian, Arawn has a terminator predicted after the major tail protein (QHJ73553.1) and a promoter located in the 3′ end of a tail protein (QHJ73556.1). As with the tail morphogenesis module of Arian and Bo-Finn, not all genes in the genomes of Arawn and Idris showed homology to previously identified proteins, but these hypothetical proteins may be involved in the tail formation. Additionally, of the predicted ORFs that were homologous to genes in other phages, 16 in the Arawn genome and 17 in Idris genome shared homology to phages of the *Siphoviridae* family, and two in each genome to phages of *Myoviridae.* Along with the absence of any tail sheath proteins which are common to *Myoviridae* (Adriaenssens and Cowan, 2014), and the majority of genes being similar to other *Siphoviridae* phages, it is likely that these two phages also belong to the *Siphoviridae* family.

Both phage Arawn and Idris were predicted confidently by PHACTS to undergo the temperate lifecycle. The major capsid proteins are similar to those in the temperate phages Streptococcus phage phiD12 and Streptococcus phage phi-SsUD.1 (Tang et al., 2013). A putative excisionase (xis) gene was predicted in both Arawn (QHJ73575.1) and Idris (QHJ73845.1), after identification of a DNA binding domain in the excisionase family. The presence of a protein homologous to a reverse transcriptase in phage Arawn (QHJ73560.1) and Idris (QHJ73832.1) suggests a possible mechanism of prophage immunity; previously shown to have an abortive effect on lytic phage infection and prolong lysogeny (Odegrip et al., 2006). Although these phages were observed *in vitro* undergoing the lytic cycle, the presence of these genes suggests the potential for Butyrivibrio phages Arawn and Idris to undergo the temperate lifecycle. The fully annotated genomes of phage Arawn and Idris can be seen in Supplementary Figures S1A,E, respectively.

As with phages Arian and Bo-Finn, a blastn search for the entire genome sequences of phage Arawn and Idris against the NCBI virus genome database did not reveal any significant alignments covering more than 8% of the query genome.

### Phage Ceridwen Genome

The longest of the Butyrivibrio phage genomes belongs to phage Ceridwen, which also has the densest genome, with more predicted ORFs in the genome proportional to the length (Table 2). Of these ORFs, ∼40% shared similarity with a viral sequence or protein in one of the databases searched. This genome shows similar organization to the other five phage genomes, with packaging, structural proteins and DNA modification related genes mostly found together (Supplementary Figure S1D). As with the other genomes, there is evidence of a tail morphogenesis module, formed of a putative tape measure protein (QHJ73746.1), tail protein (QHJ73747.1), and endopeptidase tail protein (QHJ73748.1). Of the predicted ORFs that were homologous to genes in other phages, 20 shared homology to phages of the *Siphoviridae* family, and one of *Podoviridae.* Therefore, it is likely that this phage belongs to the *Siphoviridae* family.

Phage Ceridwen was predicted non-confidently as having a lytic lifecycle by PHACTS, but the presence of an ORF similar to an antirepressor (QHJ73772.1) suggests lysogenic abilities. This antirepressor may act on the gene upstream of it; a gene homologous to the XRE family of translational inhibitors (QHJ73771.1). The most characterized phage repressors belong to this family (Durante-Rodríguez et al., 2016), which act as the main repressor, mediated by the antirepressor to trigger downstream lysis genes (Argov et al., 2019), which in this case could be the neighboring putative lysin (QHJ73773.1). Despite this, there is no evidence of excisionase genes, and this phage was only observed to undergo the lytic lifecycle *in vitro*.

A blastn search for the entire genome sequences of phage Ceridwen against the NCBI virus genome database did not reveal any significant alignments covering more than 1% of the query genome.

### Similarities and Differences in the Butyrivibrio Phages

The five phages in this study were observed *in vitro* to undergo the lytic lifecycle, infecting the same *B. fibrisolvens* host strain. The phages likely belong to the *Siphoviridae* family, as evidenced through genes encoded, genome organization, size, and similarity to other phages belonging to this family. All five phages possess genomes with sizes > 20 Kbp and <125 Kbp, and tail morphogenesis modules typical of siphoviruses (Hatfull, 2008; Veesler and Cambillau, 2011).

Despite all targeting the same bacterial host, the GC content of these phages varied, with phages Arawn and Idris having the highest GC content (46.9%), which does agree with the observation made previously that shorter phage genomes tend to have higher GC contents (Almpanis et al., 2018). There is, however, no linear relationship between GC content and genome length with these five phages (Table 2). Interestingly, phages Arawn and Idris were also predicted to be able to undergo the lysogenic cycle, yet the GC content of their genomes is higher than that of the bacterial host, which is 39.7%. These findings do not follow trends observed previously that phages either maintain a GC content similar to the host, or lower (AT rich), but may instead suggest that the phage genome integrates into an area of the host genome that is more GC rich than average (Almpanis et al., 2018).

With genome lengths of around 30–40 Kbp, this is similar to the 33.5–34.6 Kbp of other lytic rumen phage genomes belonging to *Siphoviridae* that were sequenced previously (Gilbert et al., 2017), and in the range of what has been observed from members of the *Siphoviridae* family generally (Hatfull, 2008). The number of ORFs in these Butyrivibrio phage genomes that share similarity with currently known nucleotide or amino acid sequences in the databases ranges from 40 to 52% of the total that were predicted, which is more than the ∼25–27% predicted for *Bacteroides* and *Ruminococcus* phages annotated previously, but not as many as the 54% of ORFs annotated for the *Streptococcus* phage (Gilbert et al., 2017). This lack of confidence in assigning functional annotation to all predicted ORFs stems from reliance on sequence similarity matching and poor protein characterization in phage genomes more generally. This lack of similarity to known sequences therefore is not particularly surprising, but it may suggest that the phages isolated in this study are considerably dissimilar to those isolated in other environments (Hatfull and Hendrix, 2011; Berg Miller et al., 2012). Additionally, phage genomes from the rumen are currently poorly represented in the public databases (Namonyo et al., 2018; Lawrence et al., 2019), limiting the conclusions that can be formed about the rumen phage population.

Butyrivibrio phage Bo-Finn, which was isolated from cow rumen fluid, was identified to be the same species as phage Arian, which was isolated from cow feces. This suggests that phage populations are present throughout the digestive tract and may be evidence for passage of phages that reside in the rumen through the digestive system and finally into the excretions of the ruminant animal. Whilst it is generally accepted that the fecal microbiome cannot be used to represent the rumen microbiome, but instead is more representative of the hindgut microbiota (Noel et al., 2019), in the instance of phages, sampling of feces for phages may reflect phage populations in the rumen, but this is something that requires further confirmation.

### Phylogeny Analysis Reveals Wider Context of Butyrivibrio Phages

**FIGURE 4:**
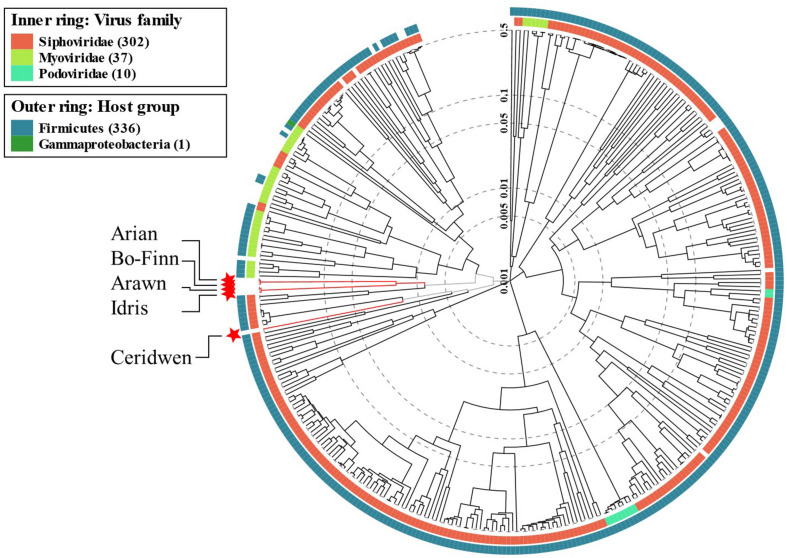
Phage phylogenetic tree. The results of the ViPTree analysis (Nishimura et al., 2017) using a protein distance metric based on normalized tBLASTx scores plotted on a log scale. The tree includes 358 related taxa, with the five Butyrivibrio phages in this study highlighted with red stars and labeled with arrows. Taxa are also annotated with the virus family they belong to (if known) on the inner ring; red for *Siphoviridae*, lime green for *Myoviridae* and turquoise for *Podoviridae*. The phyla the bacterial host belongs to for each taxa are given in the outer ring; *Firmicutes* in blue and *Gammaproteobacteria* in green. A more detailed tree with labeled taxa can be seen in Supplementary Figure S2.

There are no significant alignments to any other phage genome in the NCBI viral reference database that exceeded 8% coverage of the query genome, something that is not unusual (Hatfull and Hendrix, 2011), suggesting that these five genomes are novel. However, some predicted genes in the Butyrivibrio phage genomes did show some level of similarity to other phage genes in the databases. Phylogenetic analysis of phage proteomes using ViPTree (Nishimura et al., 2017) revealed that four of the five phages are more closely related to each other than to any other phages (Figure 4). The next closest relatives of these four phages are Lactobacillus and Paenibacillus phages. Phage Ceridwen, on the other hand, is in a clade of Clostridium phages (Supplementary Figure S2). The long branches between the Butyrivibrio phages and its nearest relatives, however, suggests low protein sequence similarity. The closest relatives to all of the Butyrivibrio phages also belong to the *Siphoviridae* family, offering further evidence that the Butyrivibrio phages belong to this family as well (Figure 4). (The full phylogenetic tree with visible labels created by ViPTree can be seen in Supplementary Figure S2).

The genomes of the 13 closest relatives of the five Butyrivibrio phages were analyzed using VICTOR to determine whether the Butyrivibrio phages, based on genome BLAST distance, belonged to an existing species, genus, or VICTOR family. It was revealed that Butyrivibrio phage Arian and Bo-Finn belonged to the same species, whilst the other Butyrivibrio phages belonged to a unique species, which was not shared with any other phage genomes. Butyrivibrio phages Arawn and Idris belonged to the same genus, which was distinct from Arian and Bo-Finn, and from Ceridwen. The Butyrivibrio phages did, however, belong to the same VICTOR family, and none of the Butyrivibrio phages belonged to the same genus or family as any other phage genomes. Phylogenomic analysis, therefore, suggests four species and three genera that are novel and unique.

### No Evidence of Phage Interactions Found in Host Genome

The reference genome for *Butyrivibrio fibrisolvens* DSM 3071 did not show an intact prophage but had two regions of incomplete prophages: one with seven predicted phage proteins of which six were highly similar to other phage proteins, and another with 10, of which six were also highly similar to other phage prophages. None of these predicted prophage proteins shared any similarity to proteins in the Butyrivibrio phage genomes from this study. Furthermore, none of the phage proteins from this study shared similarity above the thresholds to the bacterial host genome. The absence of a prophage similar to the Butyrivibrio phage genomes in this study suggests that these phages are more likely to undergo the lytic lifecycle.

Additionally, the host genome had one cas9 ortholog, and 14 CRISPR arrays, suggesting previous interaction with bacteriophages. However, none of these spacers were similar to any of the Butyrivibrio phages in this study, nor were any of the CRISPR spacers predicted in other microbial genomes in the Hungate1000 collection (Seshadri et al., 2018).

## Conclusion

This study presents five novel phage genomes isolated from rumen-associated samples, and the first phages isolated and sequenced that target *Butyrivibrio fibrisolvens.* The phages were isolated from ruminant fecal and rumen fluid samples and their genomes sequenced, analyzed, and made publicly available, a contribution that doubles the number of cultured and sequenced phages from rumen-associated samples that are publicly available. These five phages represent four novel species from three separate genera and were shown based on genomic characteristics and phylogenetic analysis to likely belong to the *Siphoviridae* family. The closest relatives based on proteome analysis to Butyrivibrio phage Ceridwen were Clostridium phages, whereas Butyrivibrio phages Arian, Bo-Finn, Arawn and Idris were more closely related to each other than to any other phage genomes.

All of these Butyrivibrio phages were observed undergoing the lytic lifecycle, and further evidence in the genomes of Arian, Bo-Finn and Ceridwen suggest that these three are virulent phages. Phages Arawn and Idris, however, harbor a number of lysogeny-associated genes, suggesting the possibility of these phages to be temperate. Yet, the lack of evidence from sequence similarity analyses of integration of these phages into the bacterial genomes of *Butyrivibrio* species does cast doubt on their lysogenic capabilities.

The addition of these five rumen-specific phage genomes to the reference databases is a small, but important contribution and will help to annotate known cultured isolates in genomic and metagenomic datasets not just from the rumen (Gilbert and Ouwerkerk, 2020), but also from other environments. There is no reason to believe that *B. fibrisolvens* would be a more or less efficient or likely phage host than any other bacterium, and the identification of five phages belonging to three different genera active against this single host suggests that there is a lot more to be discovered. However, it is likely that an effort on the international scale is required, similar to the strategies of the Hungate collection (Seshadri et al., 2018), to achieve a more representative sample of this diversity.

## Data Availability Statement

The datasets generated for this study can be found in the BioProject Accession PRJNA613207; Bo-Finn (MN882552), Arawn (MN882550), Idris (MN882554), Ceridwen (MN882553), and Arian (MN882551).

## Ethics Statement

The animal study was reviewed and approved by Aberystwyth University Animal Welfare and Ethics Review Board.

## Author Contributions

JF, AK-S, and CC conceived the project. JF carried out experimental work and analysis with support from JP, AK-S, CC, DR, and AC. All authors contributed to manuscript writing and editing.

## Conflict of Interest

DR was employed by Dynamic Extractions Ltd. The remaining authors declare that the research was conducted in the absence of any commercial or financial relationships that could be construed as a potential conflict of interest.

## Footnotes

1 https://www.aber.ac.uk/en/rbi/staff-students/ethics/animals/

2 https://www.geneious.com

3 Available at: http://s3.climb.ac.uk/ADM_share/Caudovirales.tar.gz; last modified 20/07/2017

4 Available at: http://exon.gatech.edu/GeneMark/genemarks2.cgi, accessed 10/03/2020

5 ftp://ftp.ncbi.nlm.nih.gov/blast/db/ref_viruses_rep_genomes.tar.gz; last updated 23rd August 2019

6 Release 95; ftp://ftp.ncbi.nlm.nih.gov/refseq/release/viral/

7 August 2019 release; ftp://ftp.uniprot.org/pub/databases/uniprot/current_release/knowledgebase/complete/uniprot_sprot.fasta.gz

8 v5.0, http://eggnog5.embl.de/download/eggnog_5.0/e5.viruses.faa

9 Available at ftp://ftp.ebi.ac.uk/pub/databases/Pfam/current_release/Pfam-A.hmm.gz; last modified 30/08/2018

10 Release 15, available at https://ftp.ncbi.nlm.nih.gov/hmm/TIGRFAMs/release_15.0/TIGRFAMs_15.0_HMM.tar.gz

11 HAMAP available with Prokka v1.12 (Seemann, 2014).

12 http://lowelab.ucsc.edu/tRNAscan-SE/

13 Available at http://edwards.sdsu.edu/PHACTS/upload.php; accessed on 24/03/2020

14 Available as a Galaxy tool at https://galaxy.bio.di.uminho.pt/; accessed on 24/03/2020

15 http://www.softberry.com/berry.phtml?topic=findterm&group=programs&subgroup=gfindb; version v2.8.1, accessed 24/03/2020

16 Available at: https://ggdc.dsmz.de/victor.php; accessed 09/04/2020

17 http://phaster.ca/
