# Supplementary material for: The Isolation and Genome Sequencing of Five Novel Bacteriophages From the Rumen Active Against Butyrivibrio fibrisolvens
Source: Front Microbiol. 2020 Jul 14;11:1588. doi: 10.3389/fmicb.2020.01588 (PMC7372960; doi:10.3389/fmicb.2020.01588)
Supplement: Supplementary file 1 [file Presentation_1.pptx]

## Slide 1
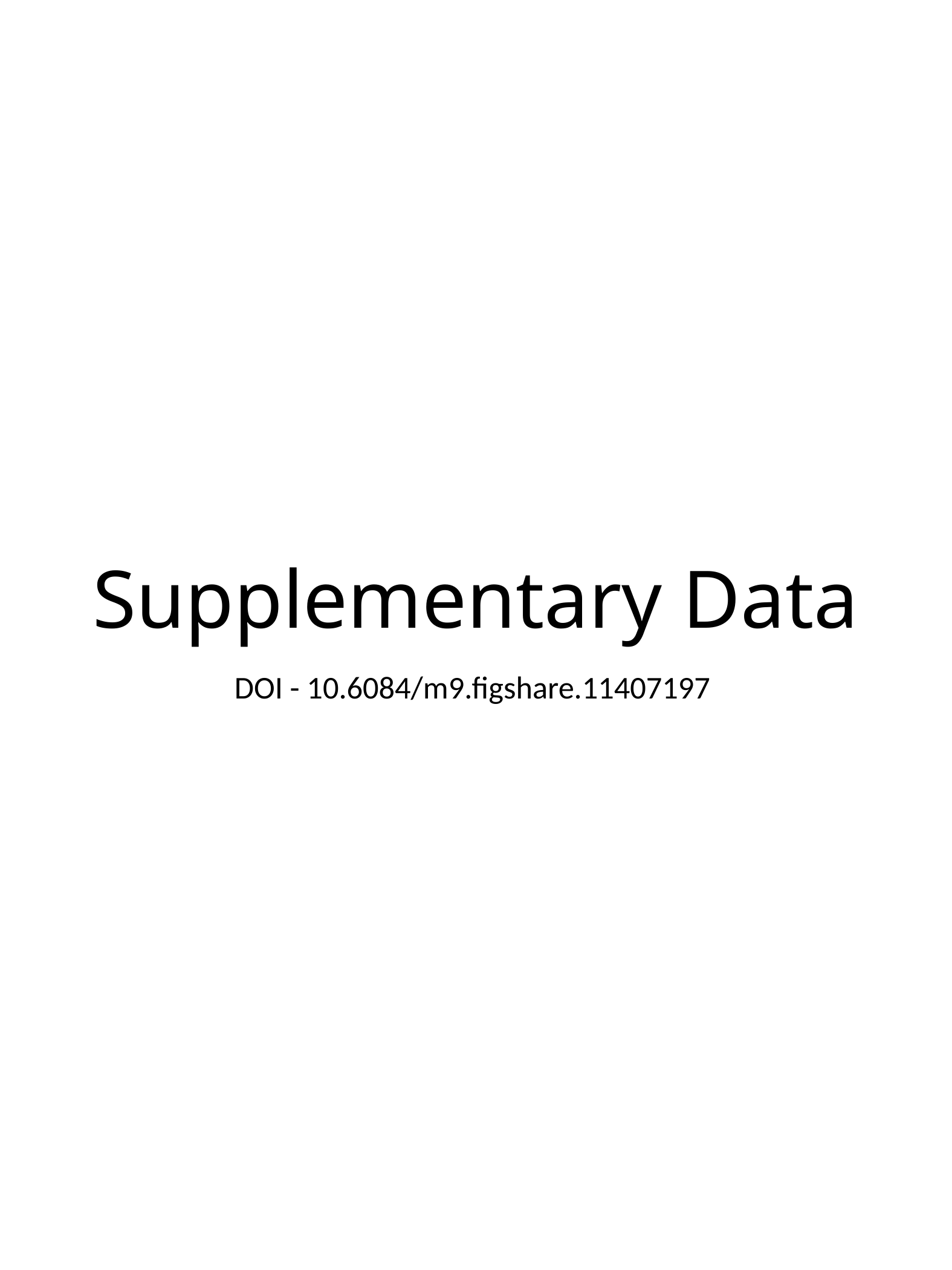

# Supplementary Data
DOI - 10.6084/m9.figshare.11407197

## Slide 2
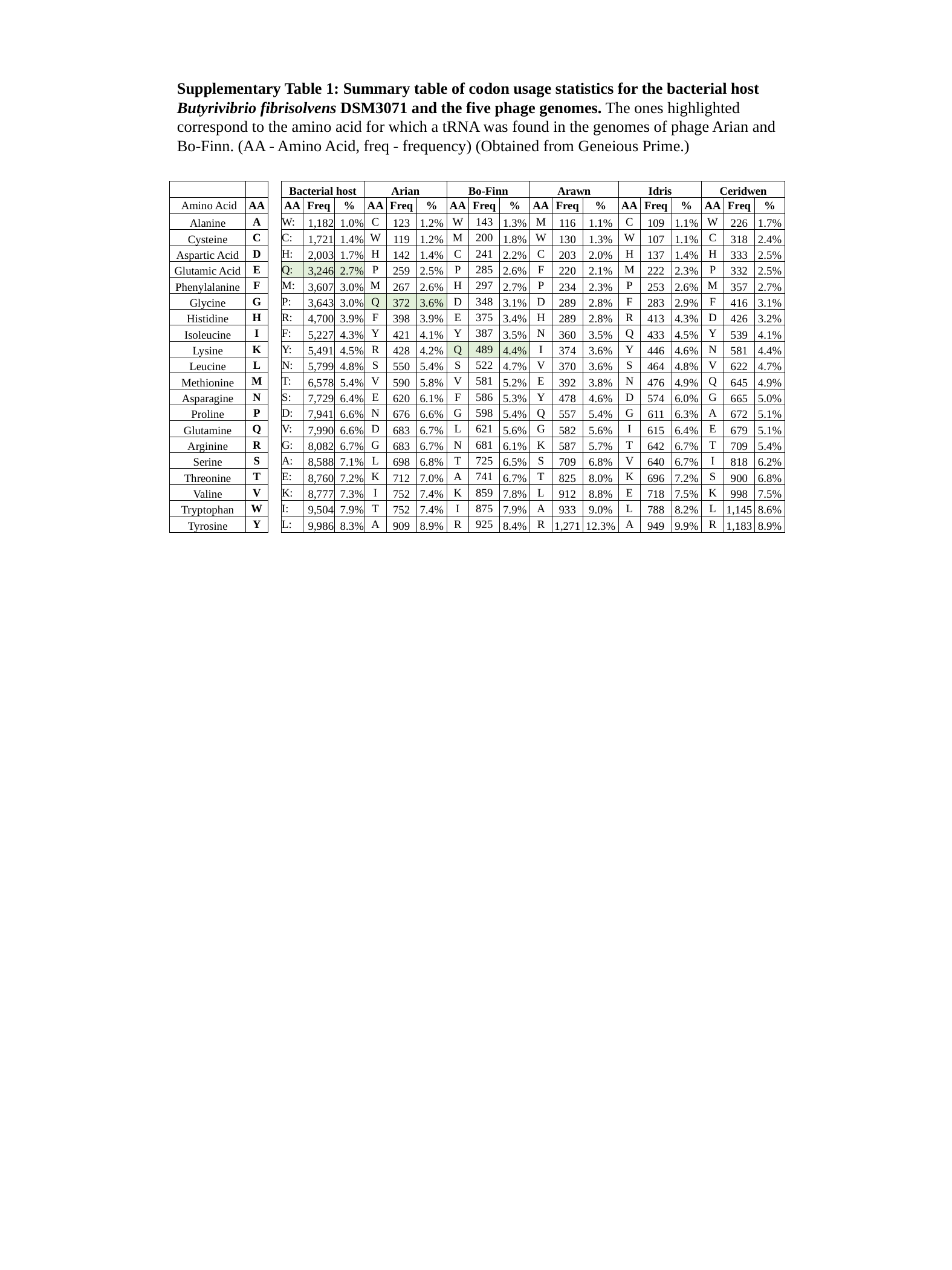

Supplementary Table 1: Summary table of codon usage statistics for the bacterial host Butyrivibrio fibrisolvens DSM3071 and the five phage genomes. The ones highlighted correspond to the amino acid for which a tRNA was found in the genomes of phage Arian and Bo-Finn. (AA - Amino Acid, freq - frequency) (Obtained from Geneious Prime.)
| | | | Bacterial host | | | Arian | | | Bo-Finn | | | Arawn | | | Idris | | | Ceridwen | | |
| --- | --- | --- | --- | --- | --- | --- | --- | --- | --- | --- | --- | --- | --- | --- | --- | --- | --- | --- | --- | --- |
| Amino Acid | AA | | AA | Freq | % | AA | Freq | % | AA | Freq | % | AA | Freq | % | AA | Freq | % | AA | Freq | % |
| Alanine | A | | W: | 1,182 | 1.0% | C | 123 | 1.2% | W | 143 | 1.3% | M | 116 | 1.1% | C | 109 | 1.1% | W | 226 | 1.7% |
| Cysteine | C | | C: | 1,721 | 1.4% | W | 119 | 1.2% | M | 200 | 1.8% | W | 130 | 1.3% | W | 107 | 1.1% | C | 318 | 2.4% |
| Aspartic Acid | D | | H: | 2,003 | 1.7% | H | 142 | 1.4% | C | 241 | 2.2% | C | 203 | 2.0% | H | 137 | 1.4% | H | 333 | 2.5% |
| Glutamic Acid | E | | Q: | 3,246 | 2.7% | P | 259 | 2.5% | P | 285 | 2.6% | F | 220 | 2.1% | M | 222 | 2.3% | P | 332 | 2.5% |
| Phenylalanine | F | | M: | 3,607 | 3.0% | M | 267 | 2.6% | H | 297 | 2.7% | P | 234 | 2.3% | P | 253 | 2.6% | M | 357 | 2.7% |
| Glycine | G | | P: | 3,643 | 3.0% | Q | 372 | 3.6% | D | 348 | 3.1% | D | 289 | 2.8% | F | 283 | 2.9% | F | 416 | 3.1% |
| Histidine | H | | R: | 4,700 | 3.9% | F | 398 | 3.9% | E | 375 | 3.4% | H | 289 | 2.8% | R | 413 | 4.3% | D | 426 | 3.2% |
| Isoleucine | I | | F: | 5,227 | 4.3% | Y | 421 | 4.1% | Y | 387 | 3.5% | N | 360 | 3.5% | Q | 433 | 4.5% | Y | 539 | 4.1% |
| Lysine | K | | Y: | 5,491 | 4.5% | R | 428 | 4.2% | Q | 489 | 4.4% | I | 374 | 3.6% | Y | 446 | 4.6% | N | 581 | 4.4% |
| Leucine | L | | N: | 5,799 | 4.8% | S | 550 | 5.4% | S | 522 | 4.7% | V | 370 | 3.6% | S | 464 | 4.8% | V | 622 | 4.7% |
| Methionine | M | | T: | 6,578 | 5.4% | V | 590 | 5.8% | V | 581 | 5.2% | E | 392 | 3.8% | N | 476 | 4.9% | Q | 645 | 4.9% |
| Asparagine | N | | S: | 7,729 | 6.4% | E | 620 | 6.1% | F | 586 | 5.3% | Y | 478 | 4.6% | D | 574 | 6.0% | G | 665 | 5.0% |
| Proline | P | | D: | 7,941 | 6.6% | N | 676 | 6.6% | G | 598 | 5.4% | Q | 557 | 5.4% | G | 611 | 6.3% | A | 672 | 5.1% |
| Glutamine | Q | | V: | 7,990 | 6.6% | D | 683 | 6.7% | L | 621 | 5.6% | G | 582 | 5.6% | I | 615 | 6.4% | E | 679 | 5.1% |
| Arginine | R | | G: | 8,082 | 6.7% | G | 683 | 6.7% | N | 681 | 6.1% | K | 587 | 5.7% | T | 642 | 6.7% | T | 709 | 5.4% |
| Serine | S | | A: | 8,588 | 7.1% | L | 698 | 6.8% | T | 725 | 6.5% | S | 709 | 6.8% | V | 640 | 6.7% | I | 818 | 6.2% |
| Threonine | T | | E: | 8,760 | 7.2% | K | 712 | 7.0% | A | 741 | 6.7% | T | 825 | 8.0% | K | 696 | 7.2% | S | 900 | 6.8% |
| Valine | V | | K: | 8,777 | 7.3% | I | 752 | 7.4% | K | 859 | 7.8% | L | 912 | 8.8% | E | 718 | 7.5% | K | 998 | 7.5% |
| Tryptophan | W | | I: | 9,504 | 7.9% | T | 752 | 7.4% | I | 875 | 7.9% | A | 933 | 9.0% | L | 788 | 8.2% | L | 1,145 | 8.6% |
| Tyrosine | Y | | L: | 9,986 | 8.3% | A | 909 | 8.9% | R | 925 | 8.4% | R | 1,271 | 12.3% | A | 949 | 9.9% | R | 1,183 | 8.9% |

## Slide 3
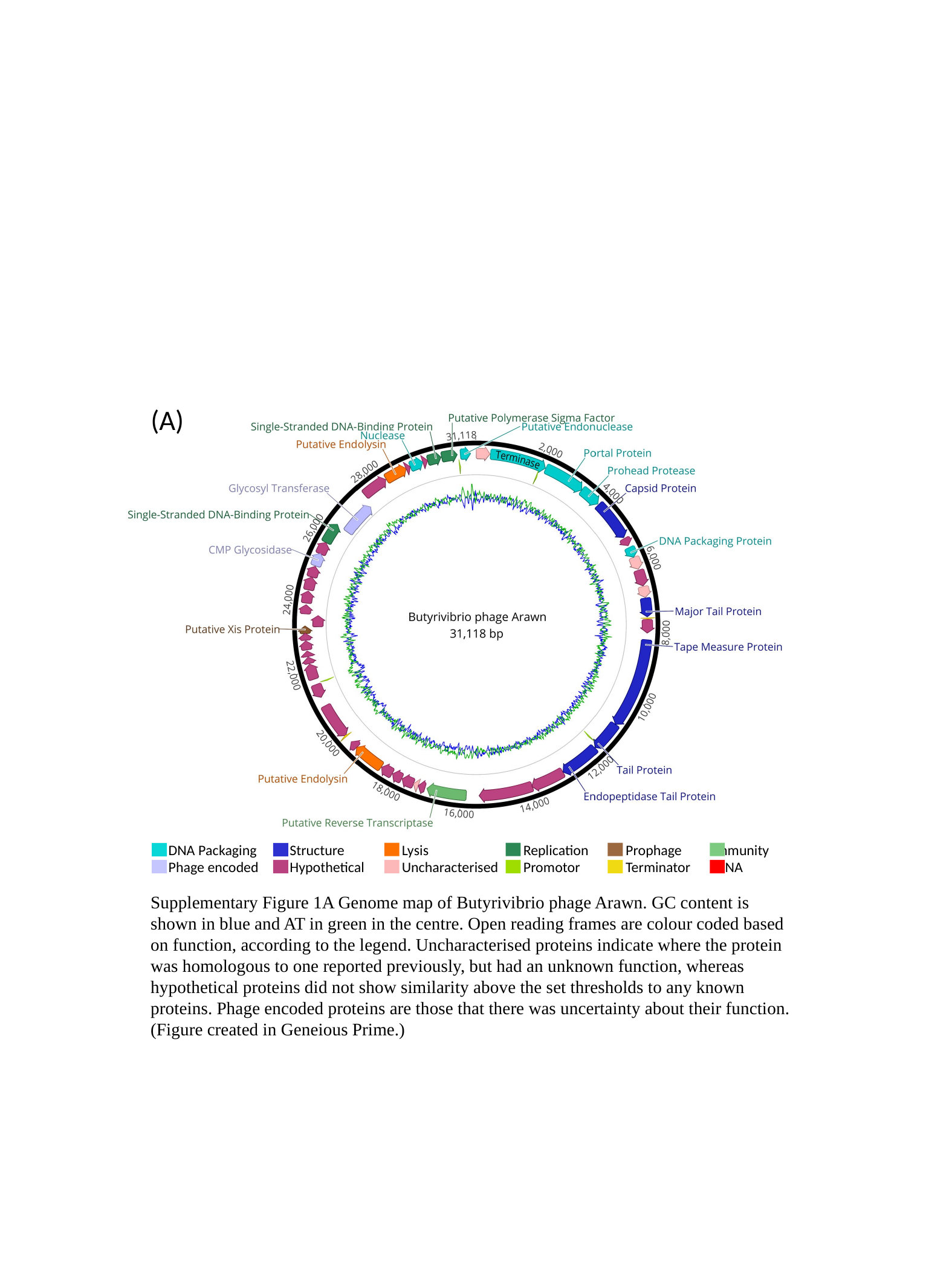

(A)
DNA Packaging	Structure	Lysis	Replication	Prophage	Immunity
Phage encoded 	Hypothetical	Uncharacterised	Promotor	Terminator	tRNA
Supplementary Figure 1A Genome map of Butyrivibrio phage Arawn. GC content is shown in blue and AT in green in the centre. Open reading frames are colour coded based on function, according to the legend. Uncharacterised proteins indicate where the protein was homologous to one reported previously, but had an unknown function, whereas hypothetical proteins did not show similarity above the set thresholds to any known proteins. Phage encoded proteins are those that there was uncertainty about their function. (Figure created in Geneious Prime.)

## Slide 4
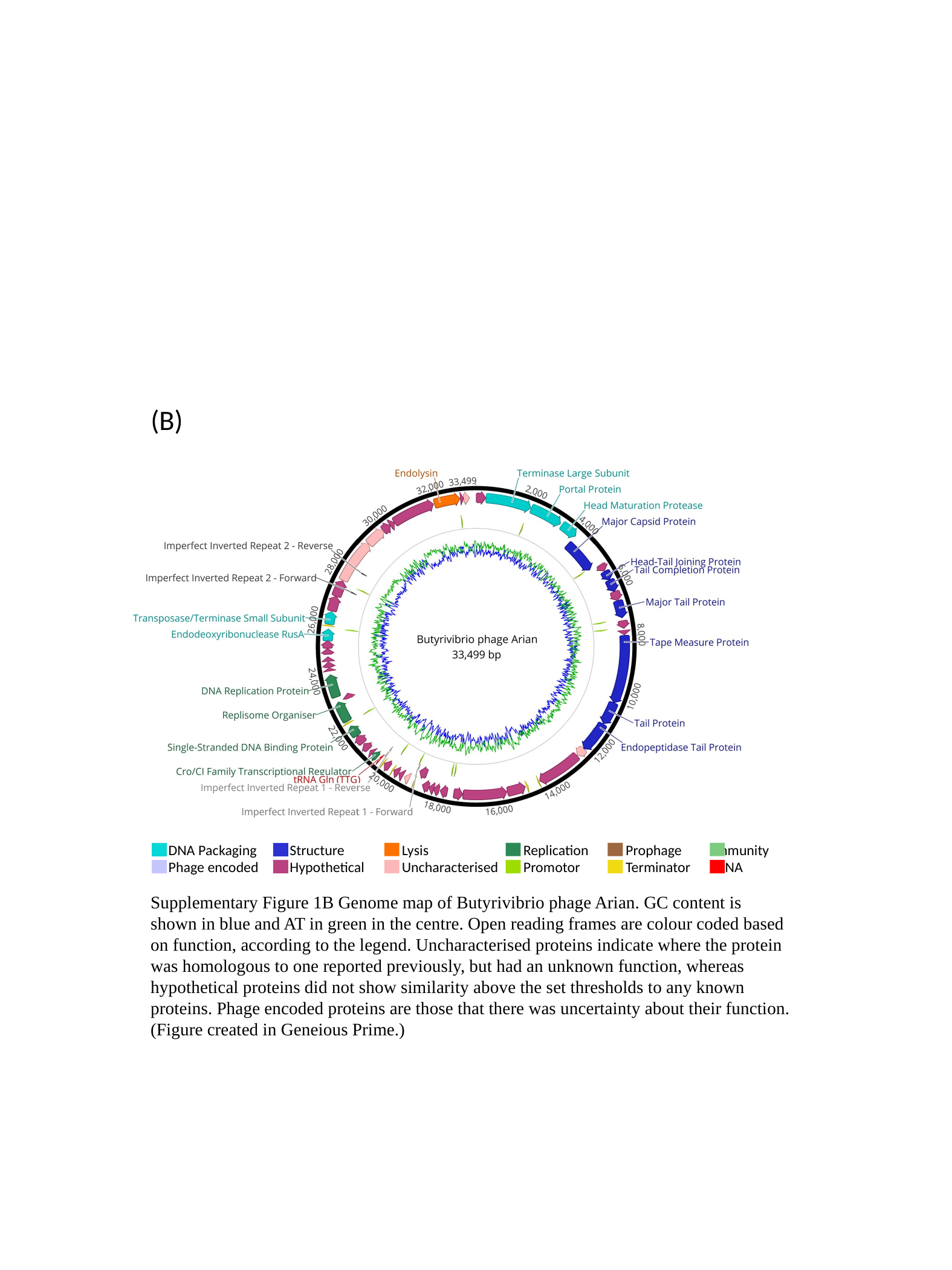

(B)
DNA Packaging	Structure	Lysis	Replication	Prophage	Immunity
Phage encoded 	Hypothetical	Uncharacterised	Promotor	Terminator	tRNA
Supplementary Figure 1B Genome map of Butyrivibrio phage Arian. GC content is shown in blue and AT in green in the centre. Open reading frames are colour coded based on function, according to the legend. Uncharacterised proteins indicate where the protein was homologous to one reported previously, but had an unknown function, whereas hypothetical proteins did not show similarity above the set thresholds to any known proteins. Phage encoded proteins are those that there was uncertainty about their function. (Figure created in Geneious Prime.)

## Slide 5
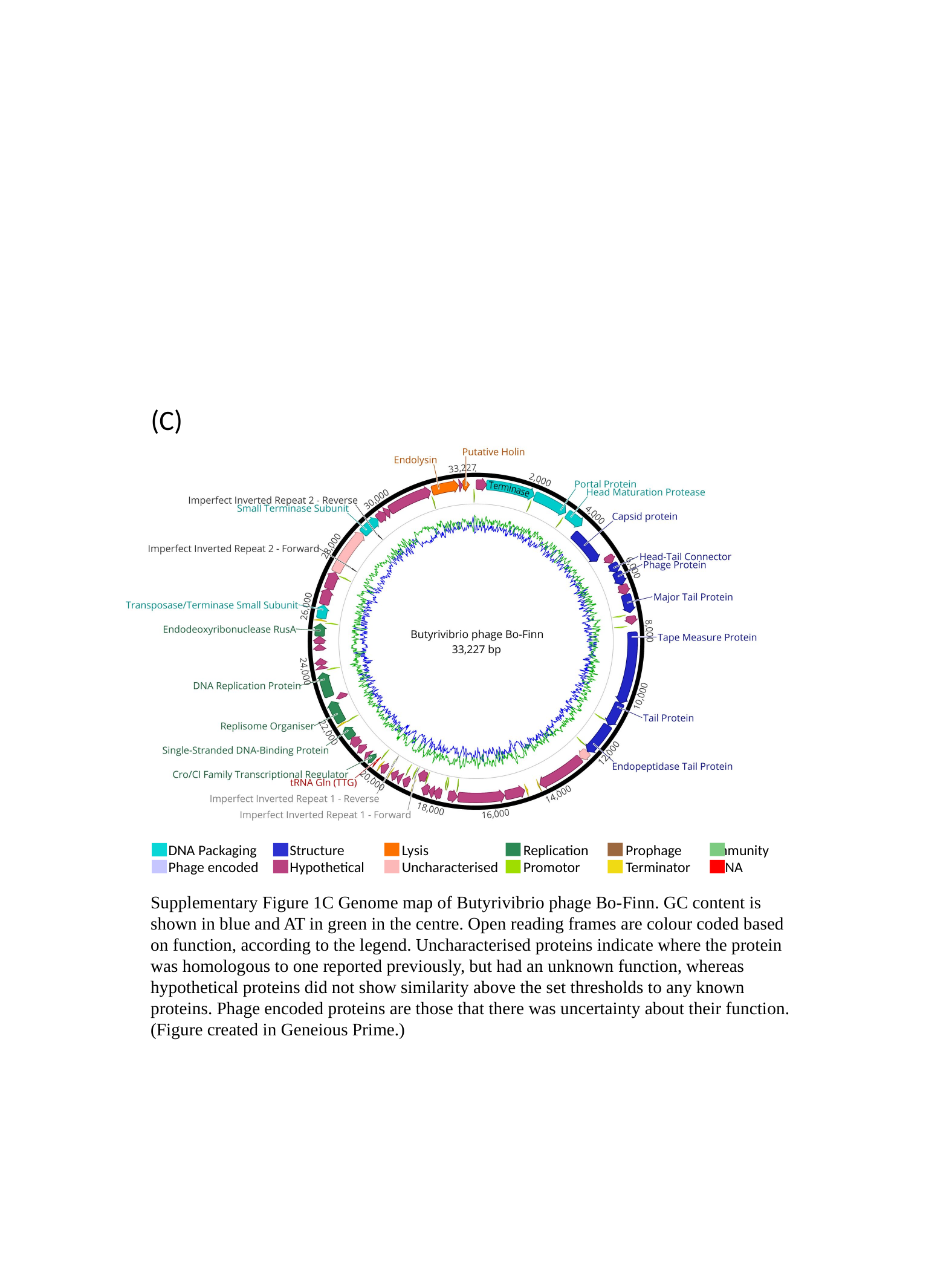

(C)
DNA Packaging	Structure	Lysis	Replication	Prophage	Immunity
Phage encoded 	Hypothetical	Uncharacterised	Promotor	Terminator	tRNA
Supplementary Figure 1C Genome map of Butyrivibrio phage Bo-Finn. GC content is shown in blue and AT in green in the centre. Open reading frames are colour coded based on function, according to the legend. Uncharacterised proteins indicate where the protein was homologous to one reported previously, but had an unknown function, whereas hypothetical proteins did not show similarity above the set thresholds to any known proteins. Phage encoded proteins are those that there was uncertainty about their function. (Figure created in Geneious Prime.)

## Slide 6
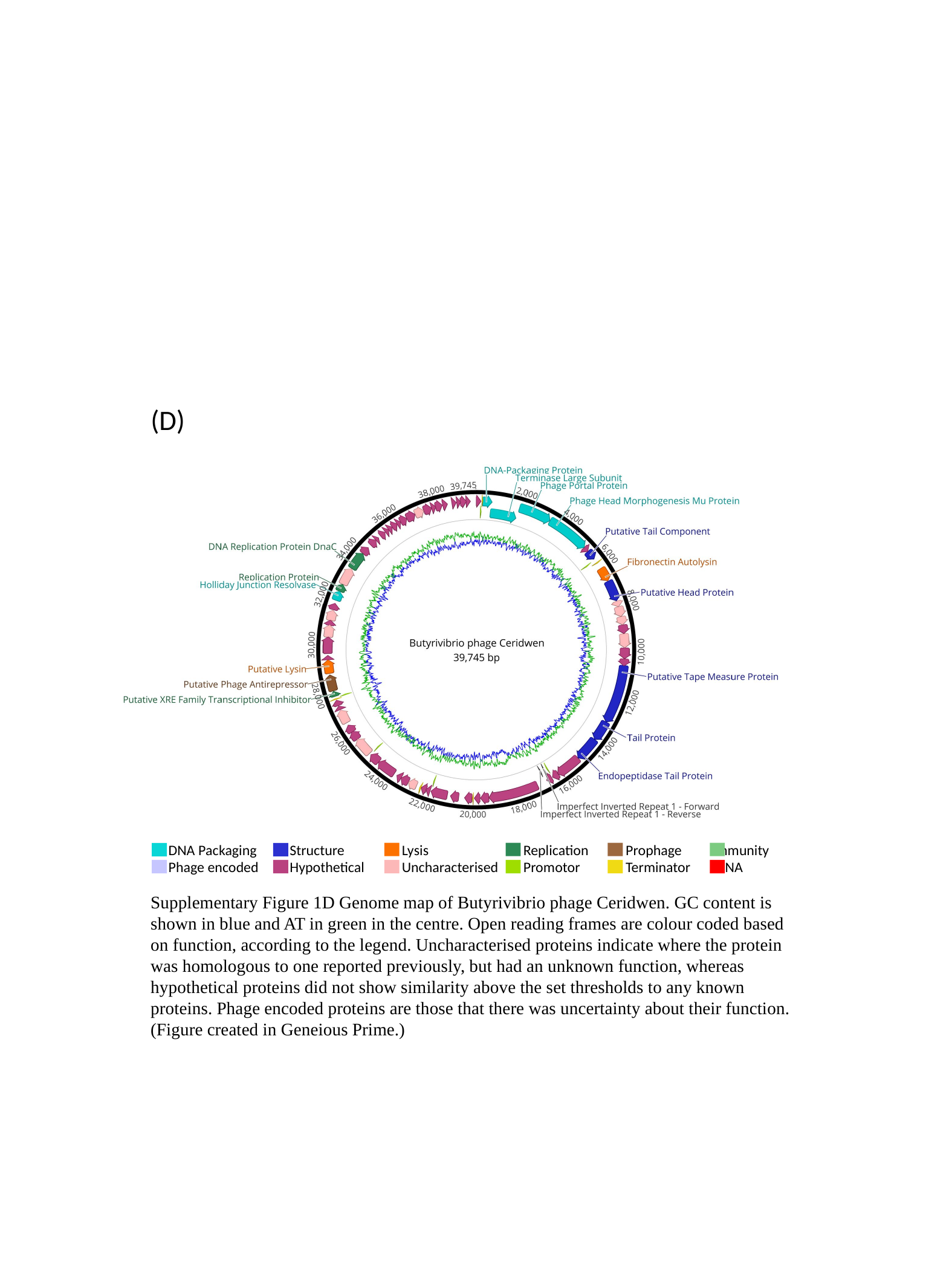

(D)
DNA Packaging	Structure	Lysis	Replication	Prophage	Immunity
Phage encoded 	Hypothetical	Uncharacterised	Promotor	Terminator	tRNA
Supplementary Figure 1D Genome map of Butyrivibrio phage Ceridwen. GC content is shown in blue and AT in green in the centre. Open reading frames are colour coded based on function, according to the legend. Uncharacterised proteins indicate where the protein was homologous to one reported previously, but had an unknown function, whereas hypothetical proteins did not show similarity above the set thresholds to any known proteins. Phage encoded proteins are those that there was uncertainty about their function. (Figure created in Geneious Prime.)

## Slide 7
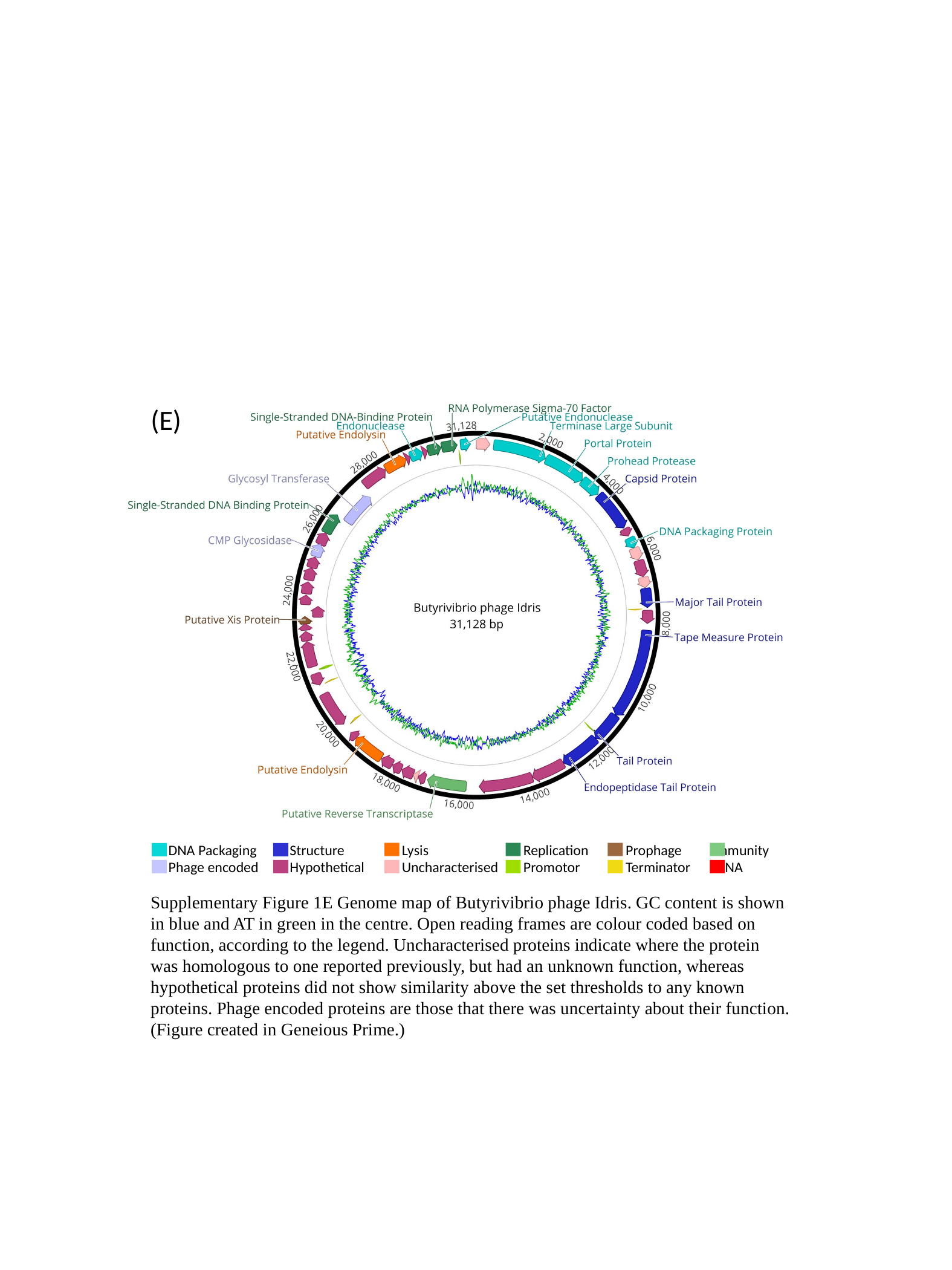

(E)
DNA Packaging	Structure	Lysis	Replication	Prophage	Immunity
Phage encoded 	Hypothetical	Uncharacterised	Promotor	Terminator	tRNA
Supplementary Figure 1E Genome map of Butyrivibrio phage Idris. GC content is shown in blue and AT in green in the centre. Open reading frames are colour coded based on function, according to the legend. Uncharacterised proteins indicate where the protein was homologous to one reported previously, but had an unknown function, whereas hypothetical proteins did not show similarity above the set thresholds to any known proteins. Phage encoded proteins are those that there was uncertainty about their function. (Figure created in Geneious Prime.)

## Slide 8
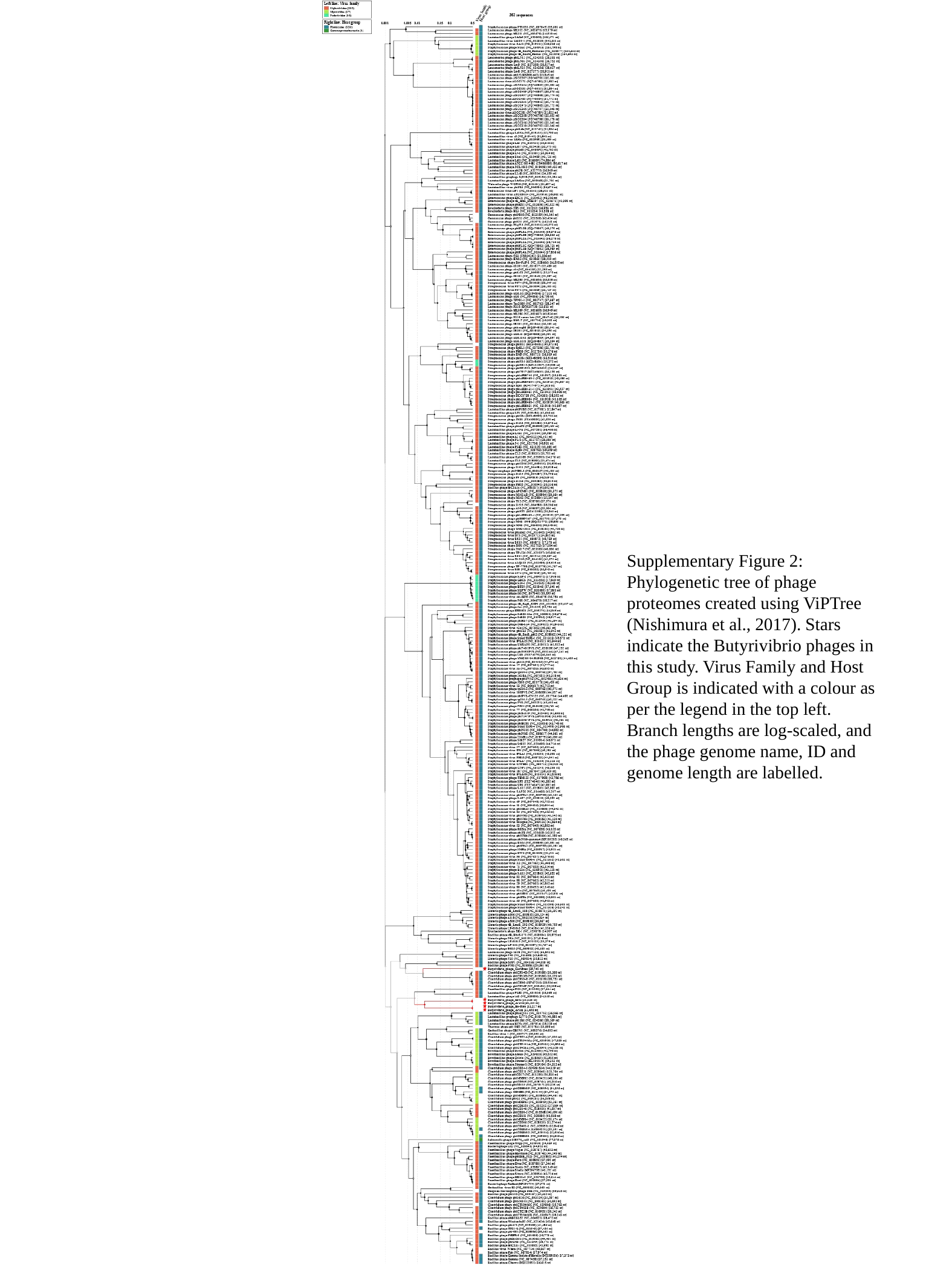

Supplementary Figure 2: Phylogenetic tree of phage proteomes created using ViPTree (Nishimura et al., 2017). Stars indicate the Butyrivibrio phages in this study. Virus Family and Host Group is indicated with a colour as per the legend in the top left. Branch lengths are log-scaled, and the phage genome name, ID and genome length are labelled.

## Slide 9
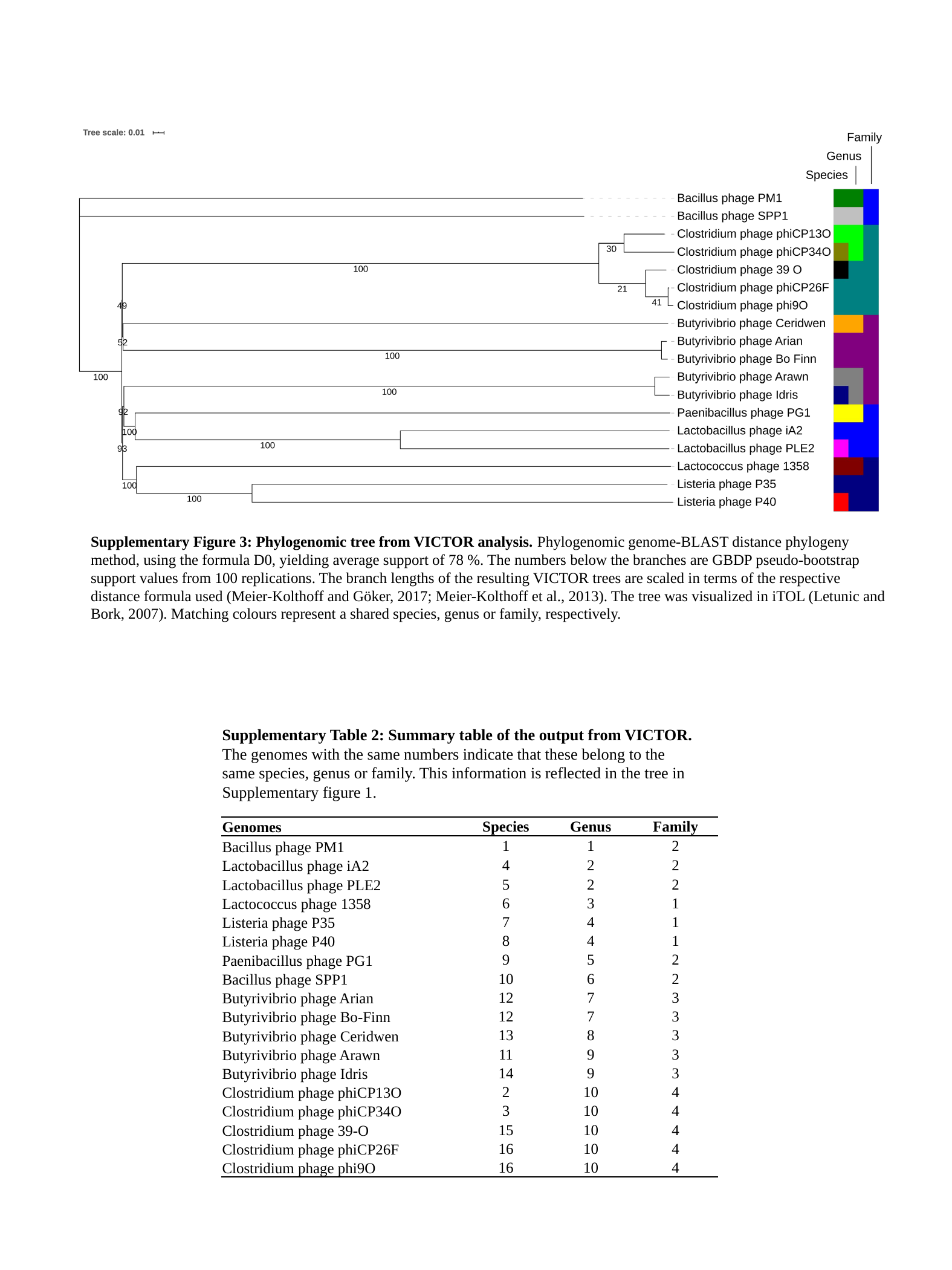

Supplementary Figure 3: Phylogenomic tree from VICTOR analysis. Phylogenomic genome-BLAST distance phylogeny method, using the formula D0, yielding average support of 78 %. The numbers below the branches are GBDP pseudo-bootstrap support values from 100 replications. The branch lengths of the resulting VICTOR trees are scaled in terms of the respective distance formula used (Meier-Kolthoff and Göker, 2017; Meier-Kolthoff et al., 2013). The tree was visualized in iTOL (Letunic and Bork, 2007). Matching colours represent a shared species, genus or family, respectively.
Supplementary Table 2: Summary table of the output from VICTOR. The genomes with the same numbers indicate that these belong to the same species, genus or family. This information is reflected in the tree in Supplementary figure 1.
| Genomes | Species | Genus | Family |
| --- | --- | --- | --- |
| Bacillus phage PM1 | 1 | 1 | 2 |
| Lactobacillus phage iA2 | 4 | 2 | 2 |
| Lactobacillus phage PLE2 | 5 | 2 | 2 |
| Lactococcus phage 1358 | 6 | 3 | 1 |
| Listeria phage P35 | 7 | 4 | 1 |
| Listeria phage P40 | 8 | 4 | 1 |
| Paenibacillus phage PG1 | 9 | 5 | 2 |
| Bacillus phage SPP1 | 10 | 6 | 2 |
| Butyrivibrio phage Arian | 12 | 7 | 3 |
| Butyrivibrio phage Bo-Finn | 12 | 7 | 3 |
| Butyrivibrio phage Ceridwen | 13 | 8 | 3 |
| Butyrivibrio phage Arawn | 11 | 9 | 3 |
| Butyrivibrio phage Idris | 14 | 9 | 3 |
| Clostridium phage phiCP13O | 2 | 10 | 4 |
| Clostridium phage phiCP34O | 3 | 10 | 4 |
| Clostridium phage 39-O | 15 | 10 | 4 |
| Clostridium phage phiCP26F | 16 | 10 | 4 |
| Clostridium phage phi9O | 16 | 10 | 4 |
